# TCMM: A unified database for traditional Chinese medicine modernization and therapeutic innovations

**DOI:** 10.1016/j.csbj.2024.04.016

**Published:** 2024-04-15

**Authors:** Zhixiang Ren, Yiming Ren, Zeting Li, Huan Xu

**Affiliations:** a School of Public Health, Anhui University of Science and Technology, Hefei, 231131, Anhui Province, China; b Peng Cheng Laboratory, Shenzhen, 518055, Guangdong Province, China

**Keywords:** Traditional Chinese medicine, Database, Knowledge graph, Deep learning, Graph neural network

## Abstract

Mining the potential of traditional Chinese medicine (TCM) in treating modern diseases requires a profound understanding of its action mechanism and a comprehensive knowledge system that seamlessly bridges modern medical insights with traditional theories. However, existing databases for modernizing TCM are plagued by varying degrees of information loss, which impede the multidimensional dissection of pharmacological effects. To address this challenge, we introduce traditional Chinese medicine modernization (TCMM), the currently largest modernized TCM database that integrates pioneering intelligent pipelines. By aligning high-quality TCM and modern medicine data, TCMM boasts the most extensive TCM modernization knowledge, including 20 types of modernized TCM concepts such as prescription, ingredient, target and 46 biological relations among them, totaling 3,447,023 records. We demonstrate the efficacy and reliability of TCMM with two features, prescription generation and knowledge discovery, the outcomes show consistency with biological experimental results. A publicly available web interface is at https://www.tcmm.net.cn/.

## 1 Introduction

TCM is an empirical science based on thousands of years of clinical experience and remains essential in diagnosing and treating diseases [1]. Recent investigations [2], [3], [4], [5] have demonstrated that TCM and modern medicine have a common theoretical basis at the molecular level. Specifically, compounds can effectively treat diseases by regulating the efficacy of certain targets. However, there is a lack of current knowledge regarding the chemical components and metabolism mechanisms of TCM, which obscures the association between drug components and pharmacological effects, therefore impeding targeted TCM diagnosis and treatment. Therefore, in the modernization process of TCM, it is crucial to develop a comprehensive and highly reliable TCM modernization database, which would facilitate the identification of active ingredients and clarify the action mechanism with biological targets.

Recently, numerous efforts [6], [7], [8], [9] have been conducted to construct TCM databases that incorporate knowledge on herbs, ingredients, and targets to support related research. However, these works typically overlook the critical concepts of “symptom” and “prescription” inherent in the theoretical foundation of TCM. SymMap [10] integrates symptom data, which is beneficial for drug discovery research based on phenotypes. LTM-TCM [11] and CPMCP [12] introduce the concept of prescription, enabling Chinese medicine practitioners to interpret the compatibility of herbs in prescriptions. TCMBANK [13] integrates extensive data on herb, ingredient, disease, and target entities but lacks crucial concepts such as prescription and symptom. Despite these advancements, current integrative works have only partially involved modern medical concepts like disease, target, and ingredient. This simplifies the principles of ingredient-based disease treatment and ignores essential information such as anatomy, and pathway, hindering a thorough understanding of pharmacological effects. Additionally, existing databases primarily feature binary relations, overlooking the complexity of biological interactions. Various relations may indicate the alleviation or aggravation of diseases, such as ingredient-downregulate/upregulate-target.

TCM knowledge discovery encompasses various challenging tasks such as investigating the chemical mechanisms of herbs and identifying the therapy patterns of TCM prescriptions. Although some researchers have utilized network pharmacology to analyze the mechanism of action of prescriptions [14], [15], [16] and explore Herb-Symptom correlations [17], these efforts have concentrated on analyzing specific prescription or diseases rather than developing a universal method for clarifying the mechanism. Recent progress in artificial intelligence (AI) has enabled the development of methods that integrate TCM data with deep learning (DL) for tasks like Prescription-Target interaction [18] and Herb-Symptom correlation [19]. However, the pharmacological principle of TCM remains unclear due to the absence of details, leaving a gap in the knowledge discovery of modernized TCM.

Symptom-based prescription is crucial in TCM treatment, and the integration of prescription generation with AI methods has become widely used, offering convenience for patients and practitioners. Recently, [20], [21] proposed the topic model for prescription generation that includes background knowledge such as herbal compatibility, aiming to facilitate the creation of prescriptions and integrate TCM theories into the generation process. To generate prescriptions that align more closely with TCM concepts, [22], [23], [24] concentrate on the use of Seq2Seq structures, by viewing TCM prescription generation as a sequence process task and decode the latent representation of symptoms into herb sequences. Additionally, GNN can effectively capture both structural and semantic information between entities and is commonly used in prescription generation tasks. Many studies [25], [26], [27], [28] have utilized GNN to capture correlations among herbs, symptoms, and prescriptions, recommending high-quality herb sets based on background knowledge. [29], [30], [31] employ the knowledge graph to introduce more diverse information, such as herb properties, to obtain more reliable representations. However, existing methods overlooked internal prescription properties such as the compatibility principle, while also lacking the use of mechanisms like pathway and target.

In this work, we introduce TCMM, a unified database for TCM modernization that integrates 6 high-quality databases of TCM and modern medicine. According to Table 1, TCMM is the currently largest non-commercial database for TCM modernization, consisting of 20 typical entities such as prescription, ingredient, target and 46 relations among them, aiming to comprehensively encompass the theoretical foundations of TCM and modern medicine. A web-based interface is provided for users to explore relations among medical knowledge. Based on the constructed database, a prescription generation pipeline with pre-train method is proposed, which can create highly credible prescriptions, thereby revealing the potential of the database. This pipeline is integrated into the website to support user-customized prescription generation. Furthermore, in the absence of direct data, we pioneering use a multi-hop reasoning method to achieve TCM knowledge discovery. This includes 2 challenging tasks, prescription repositioning and symptom-related target prediction. The results highly match experimental conclusions in case studies, effectively supporting TCM modernization.

**Table 1:** **TCM Database Comparison** provides a quantitative comparison of the data scale of TCMM with existing TCM databases. To ensure fairness, we only count the entity types present in existing databases. The values are based on the most recent data from the relevant databases. The results show that TCMM contains the most comprehensive entity type and the largest amount of data.

Database	Prescription	Herb	Ingredient	Target	Disease	MM Symptom	TCM Symptom	Syndrome	Pathway	Total
TCM-ID [32]	7443	2751	7375	2756	4111	/	/	/	/	24658
CHEM-TCM [8]	/	240	7000	78	/	/	/	/	/	7318
TCM@TAIWAN [7]	/	453	20000	/	/	/	/	/	/	20453
TCMSP [6]	/	502	13729	3339	867	/	/	/	/	18437
TM-MC 2.0 [9]	5075	635	34061	13991	27997	/	/	/	/	81759
HerDing [33]	/	**19476**	6655	16762	11394	/	/	/	/	54287
TCMID 2.0 [34]	46929	8159	43413	17603	4633	/	/	/	/	65649
ETCM [35]	3959	402	7284	2266	4323	/	/	/	/	18234
SymMap v2 [10]	/	698	25975	20965	14086	1148	**2285**	**233**	/	65390
HITv2 [36]	/	1250	1237	2208	/	/	/	/	/	4695
HERB [37]	/	7263	49258	12933	28212	/	/	/	/	97666
CPMCP [12]	2125	1560	27928	20965	14434	1148	2285	/	/	70445
LTM-TCM [11]	**48126**	9122	34967	13109	/	/	1928	/	/	107252
TCMBank [13]	/	9191	61965	15179	**32529**	/	/	/	/	118864
**TCMM**	48043	8932	**69816**	**76449**	22365	**17079**	1900	146	**3704**	**248434**

## 2 Materials and methods

### 2.1 Data collection and processing

#### 2.1.1 Data source and statistics of TCMM

**Fig. 1:**
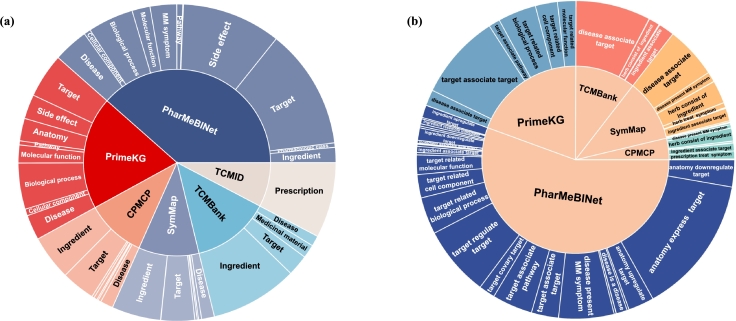
**Data Source of TCMM Database** provides a visual representation of the data sources of entities and relations in TCMM. The size of each area represents the quantity of entities or relations in that source. **(a), (b) Data Source of TCMM Entities and Relations** integrates modern medical knowledge from PharMeBINet, PrimeKG and integrates TCM knowledge from TCMBank, CPMCP, and SymMap. Specific rules and LLM are used for data integration.

**Fig. 2:**
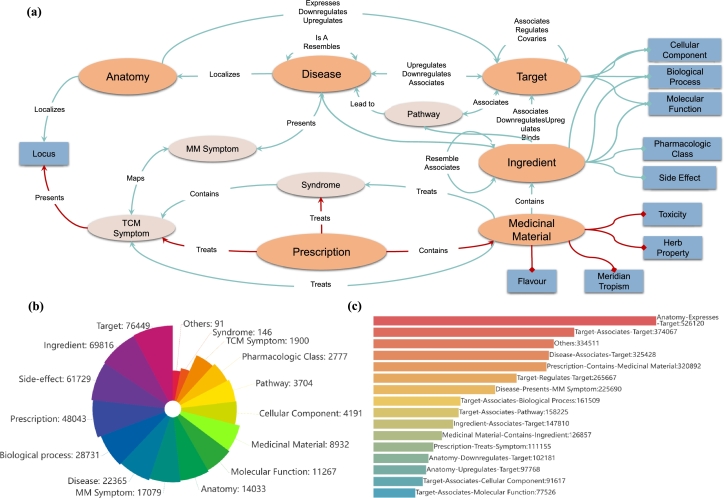
**Overview of TCMM Database.(a) Details of TCMM Database** displays the complete 20 entities and 46 relations in the database, while the blue bars represent the attribute entities and the red lines represent the novel relations. **(b) Entity Distribution in TCMM** provides a quantitative overview of the entity types in TCMM. TCM attribute entities are a small minority and are therefore grouped as “others”. **(c) Relation Distribution in TCMM** illustrates the quantity of each relation type within the TCMM. 31 types of relations, due to their low frequency, have been collectively categorized as ‘others’.

TCMM aggregates knowledge from six leading databases across the domains of TCM and modern medicine, which are PrimeKG [38], PharMeBINet [39], TCMBank [13], SymMap [10], CPMCP [12] and TCMID [34]. This integration has constructed a robust knowledge network, including 248,434 entities and 3,447,023 relational triplets. By aligning the TCM and modern medical data, TCMM yields relations with enhanced detail and broadens the diversity of entity types encompassed within the network. Fig. 2 illustrates the comprehensive scope of TCMM, showcasing 20 distinct types of entities and 46 varieties of relations. The data sources integrated into TCMM are shown in Fig. 1, ensuring transparency and accessibility for further research and analysis.

In this work, entities related to TCM symptoms, medicinal materials, prescriptions, and other TCM knowledge are gathered from CPMCP, SymMap, TCMID and TCMBank. By collecting herb information from https://zhongyibaike.com, we standardize the nomenclature of medicinal materials and harmonize the names of the herbs within the prescriptions. The CPMCP and SymMap databases serve as the primary sources for TCM symptoms. Due to the semantic overlap among symptoms, a large language model (LLM) employing instruction learning is utilized to measure the semantic distances between symptom representations, facilitating the amalgamation of synonymous symptoms. This process is further validated through manual verification to ensure the reliability of the results. For prescription, data from TCMID is employed to enrich the entities included in the CPMCP database.

Modern medical data are primarily derived from PharMeBINet and PrimeKG. Nevertheless, certain entities require merging TCM databases with modern medical databases by aligning and cross-referencing information to resolve differences in External IDs across various resources. For instance, the utilization of SymMap ID facilitates the alignment of ingredients within SymMap, CPMCP, and TCMBank. Records in TCMBank may refer to multiple SymMap IDs, which are excluded. PharMeBINet uses ingredient names for alignment since SymMap IDs are not available. Identifiers like name, MONDO ID, OMIM ID, MeSH ID, Orphanet ID, and UMLS ID are collectively utilized to synchronize diseases across databases. Notably, phenotypes classified as diseases in TCMBank, such as ‘height’ and ‘cardiovascular issues’, are manually excluded. Additionally, properties including biological process, cellular component, molecular function, pharmacologic class, and side effect are integrated to enhance comprehension of the mechanisms of action and potential risks associated with herbal medicines. The supplementary table S1 contains information on the source, number, and integration approach of all entities.

#### 2.1.2 Relation processing among TCMM entities

This study establishes 8 novel relations among the components, leading to the introduction of new perspectives. Notably, the Prescription-Syndrome, a pioneering contribution of this research that has not been previously documented in any publicly available databases, is introduced by correlating syndromes with symptoms. Properties including locus, tropism, flavor, property, and toxicity, typically presented in an unstructured format in public databases, are systematically arranged through text recognition to eliminate ambiguities while establishing five relations. The relations of Prescription-Symptom and Prescription-Medicinal material, sourced from the CPMCP database, initially include only 2,279 prescriptions. To expand the number of prescriptions, we employ the LLM to extract prescription descriptions from the TCMID database, enriching the database with an additional 31,678 prescriptions for Prescription-Symptom and 44,447 for Prescription-Medicinal material. Moreover, this effort is the first to extract herb proportions from prescriptions, supporting research in dosage prediction and compatibility principles. Supplementary table S2 details the source, quantity, and integrating strategy for each relation, providing a comprehensive overview of the data integration methods used in this work.

*Large language model based knowledge extraction*

Prescriptions in public databases are primarily collected from ancient documents, with descriptions typically in the unstructured format, and there is a lack of consistency in terms of symptoms and medicinal dosages. For example, the medicinal dosage is characterized by a variety of measurement units such as ‘liang’, ‘qian’, ‘zhu’, and ‘fen’. To address this issue, a data processing pipeline based on LLM is developed to intelligently arrange the relations of Prescription-Symptom and Prescription-Medicinal material within the text. The entire workflow is illustrated in supplementary figure S1.

The process for Prescription-Symptom begins with gathering TCM symptoms and properties from CPMCP, as seen in supplemental figure S1 (a). To unify overlapping semantics of TCM symptoms, semantic descriptions are initially generated through prompt engineering and GPT methods [40], [41], subsequently transformed into embedding representations with Sentence BERT [42]. Symptoms are classified based on locus and property to improve computing efficiency, with cosine similarity computations confined within each group. A similarity threshold of 0.98, determined through manual verification, enables the merging of symptoms preceding this threshold into a single record, resulting in a unified list of 1,900 TCM Symptoms. This list serves as a dictionary, mapping merged information (key) to semantically similar symptoms (value), which is subsequently utilized to match symptom descriptions in prescriptions.

For Prescription-Medicinal Material, keyword matching is initially employed with herbs from CPMCP to extract preliminary Prescription-Medicinal Material triplets from prescription descriptions. The herb names in these triplets are standardized using information from www.zhongyibaike.com. According to the supplementary figure S1 (b), ChatGLM [43] is utilized to obtain details about medicinal materials and their dosages from prescription descriptions. Due to the initial precision limitation, a manually annotated medicinal material-dosage dataset is used to finetune ChatGLM, which significantly improves accuracy and recall. Rule matching provides an additional prediction method to enhance accuracy for prescriptions that are wrongly predicted. Furthermore, to modernize TCM dosage, only 17 prevalent weight/volume units are retained, with ancient or imprecise units converted to grams or milliliters. The process for dosage unit conversion is explained in supplementary table S3.

*Rule based knowledge correction*

The semantic differences among relations in public databases present a significant challenge, requiring rule-based alterations to align variances across diverse resources. Our method begins by involving a series of exclusionary rules aimed at addressing the complex relations in modern medicine. For instance, the relation between Ingredient and Target can manifest in forms such as Associates, Downregulates, Upregulates, and Binds. Given the semantic opposition between Downregulates and Upregulates, We hypothesize that these relations should not concurrently apply to the same entity pair. Moreover, considering the ambiguity of the Associates relation, it is considered mutually exclusive with all definite relations to reduce redundancy.

Furthermore, relations of Associates, Covaries, and Resembles are identified as undirected but are represented in a directed format within our database. For example, TCMM specifies that when reciprocal representations exist (e.g., ‘A – Associates – B’ and ‘B – Associates – A’), only one directed instance (‘A – Associates – B’) shall be preserved to maintain clarity and consistency. The details of these processing rules are available in supplementary table S2.

### 2.2 Knowledge augmentation based TCM prescription generation

**Fig. 3:**
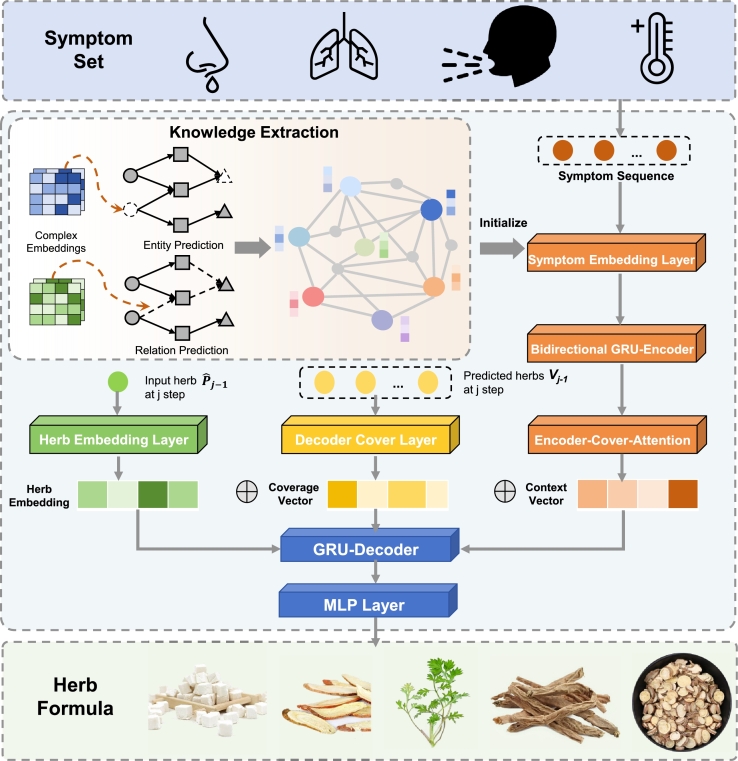
**TCM Prescription Generation Model. Knowledge Extraction** employs the ComplEX [44] model to treat ‘knowledge graph completion’ as a pre-training task, facilitating the integration of TCMM knowledge. The model first initializes the entities and relations in the knowledge graph to the complex space to acquire complex embeddings. Then, it conducts collaborative model training on two tasks: entity completion and relation completion. The pre-trained entity embedding is then used as the initial parameter of the symptom embedding layer. **Prescription Generation** model adopts a Seq2Seq architecture, mapping the input symptom set into a herb formula. Bidirectional GRU is utilized as the Encoder to map the pre-trained symptom embeddings into a context vector. The Decoder then progressively decodes the context vector into the herb ID *P*_*j*_. To prevent the generation of duplicate herbs, a multi-hot vector *V*_*j*_ is introduced to annotate the predicted herbs.

In this study, we implement a TCM prescription generation pipeline upon the work proposed by [45], which grounds in the Encoder-Decoder framework shown in Fig. 3. By combining it with the Diverse Beam Search [46], the model is proven to be capable of producing more diverse prescriptions. Furthermore, we extract relevant triplets from the database to construct a KG and incorporate Chinese and Western medical knowledge into the model via a graph pre-train task. Experimental results demonstrate that database knowledge enhances metrics and leads to more reasonable prescriptions.

#### 2.2.1 Data processing

Prescription-Symptom pairs from TCMM are used as the dataset from which to develop a model that generates sequences from symptom to herb. To unify the order of sequence data, symptom and medicinal material sequences are sorted according to ID in the database. The dataset encompasses a total of 32,937 prescriptions, including 1,820 distinct symptoms and 1,682 types of medicinal materials. On average, each prescription integrates 7.09 medicinal materials and correlates with 3.32 symptoms. To facilitate the development and evaluation of models, these prescriptions have been randomly allocated into training, validation, and testing subsets following an 8:1:1 ratio.

To clarify the relation between herbs and symptoms, the model's initial parameters employ embeddings pre-trained on a KG. Expert knowledge directs the extraction of prescription generation-related triplets from the TCMM database, constructing a KG encompassing entities such as herb, prescription, ingredient, target, TCM symptom and MM symptom. The KG structure is depicted in supplementary figure S2.

#### 2.2.2 Model description

To capture the long-term dependency in symptom sequences, the GRU [47] is utilized as the Encoder to transform variable-length symptom sequences ($s_{1}$, ..., $s_{M}$) into hidden state sequences ($h_{1}$, ..., $h_{M}$). Additionally, the bidirectional GRU is employed to adapt to the weak order characteristics of symptom sequences, capturing symptoms before and after the current moment. The hidden state $h_{t}\in R^{2⁎hs}$ at time t consists of the forward state $h_{f}\in R^{hs}$ and the reverse state $h_{r}\in R^{hs}$, with the calculation process as follows:

(1) $$h_{t}=h_{f}⊕h_{r}\;h_{f}=F_{f}\left(h_{t-1},E_{st}\right)h_{r}=F_{r}(h_{M-t}^{\prime },E_{st})$$

In equation (1), $E_{st}$ represents the embedding of $s_{t}$, while $F_{f}$ and $F_{r}$ are the forward GRU and reverse GRU networks, respectively. $h_{M-t}^{\prime }$ is the reverse symptom sequence.

The attention mechanism is utilized to enable the decoder to better model long sequence information of symptoms. Specifically, based on the previous state $s_{j-1}$ and attention $A_{j-1}$ of the symptom, the weight of the symptom hidden state at the current moment $\alpha_{\mathrm{tj}}$ is measured, yielding the context vector $c_{j}$ for the current moment of the decoder. The function a is a soft alignment function used to compute the correlation between s and h.

(2) $$c_{j}=\sum_{t=1}^{T}\alpha_{\mathrm{tj}}h_{t}\;\alpha_{\mathrm{tj}}=\mathrm{softmax}\;\left(a\left(s_{j-1},{\;h}_{t},A_{j-1}\right)\right)$$

The Decoder utilizes the GRU to decode the hidden state of symptoms into a variable-length herbal sequence incrementally. Since the prescription generation task aims to generate a non-repetitive herbal sequence, a Cover layer is introduced to make the model aware of generated tokens, thereby generating a more diverse and reasonable herbal formula. The predicted herbs are transformed into a multi-hot vector representation $V_{j-1}$, which is subsequently projected onto the hidden space $CV_{j-1}$ via the Cover layer. This, in conjunction with the context vector $c_{j}$, the previous state $s_{j-1}$, and the herb embedding in the previous timestep $E_{h}j$, is collectively used to update the current state $s_{j}$ of the Decoder. The MLP then decodes $s_{j}$ into the herb ID $p_{j}$.

(3) $$p_{j}=\mathrm{softmax}\;\left(W_{\text{out }}^{T}s_{j}+b_{\text{out }}\right)s_{j}=F_{\mathrm{Decoder}}\left(s_{j-1},\mathrm{Concat}\;\left[c_{j};E_{hj};{\mathrm{cv}}_{j-1}\right]\right){\mathrm{cv}}_{j-1}=\tanh\left(W_{\mathrm{cover}}^{T}V_{j-1}+b_{\mathrm{cover}}\right)$$

Pre-trained embedding is introduced as the initial parameter for our model to enhance the performance of downstream tasks. To capture the semantics within the complex graphical structure, we design a KGC task, which aims to explore high-order correlation and encode the intricate information in heterogeneous graph. ComplEX [44] is utilized as the pre-trained model since its outstanding performance in the KGC task. According to [44], in order to more fully integrate the knowledge within the graph, both entity prediction and relation prediction are jointly used as training tasks.

(4) $$\arg\max\sum_{\langle u,r,v\rangle \in G}\log p(u|r,v)+\log p(v|u,r)+\lambda \log p(r|u,v)$$

As the equation (4) shows, the objective of ComplEX is to maximize the predictive probability of the combined optimization equation, encompassing three tasks: predicting the head entity u, the tail entity v, and the relation r.

Cross entropy is used as the loss function of the model. However, the original cross entropy function requires that the target sequence have a strict order to measure the loss of the predicted sequence. Since the herb sequence in the prescription generation task does not demand a strict order, this work employs the smoothed target probability $y_{j}^{\prime }$ to relax the penalty for incorrect prediction of herb position.

(5) $$y_{j}^{\prime }=\left(y_{j}+g/T\right)/2\;\mathrm{loss}=-\sum_{j}\sum_{vs}y_{j}^{\prime }\log\left({\overset{ˆ}{p}}_{j}\right)$$

${\overset{ˆ}{p}}_{j}$ is the predicted probability that the herb appears at the jth position of the prescription. $y_{j}$ is the one-hot encoding of the herb at the jth position of the real-world prescription sequence. g is the multi-hot encoding of the herbs in the real-world prescription. If the herb is present in the prescription, the element at the corresponding position in the g is set to 1, otherwise to 0. T is the length of the real-world prescription and vs represents the vocabulary size.

To support the online inference, it is essential to ensure that the result is globally optimal and has a lower time complexity. Diverse Beam Search [46] strikes a balance between greedy search and exhaustive traversal, dividing the search space into G Beam Search groups, retaining the B sequences with the highest probability as candidates in each iteration, and introducing diversity penalty to enhance sequence diversity.

#### 2.2.3 Experimental study

Two comparative experiments are conducted to evaluate the performance of the prescription generation models. The first set aims to evaluate the performance between models integrating TCMM knowledge and baseline models. The second set is designed to assess the enhancement resulting from the incorporation of different types of modernized TCM knowledge into the prescription generation model. The following models are chosen for comparison:

**TCMPR**[28]: This model utilizes a Herb-Symptom related knowledge graph. It extracts a subnetwork of symptoms from the knowledge graph, effectively representing the characteristics of symptoms, particularly terms not documented in clinical settings.

**KDHR**[31]: A graph convolution model that utilizes herb attributes as supplementary knowledge. In this work, we leverage herb-related properties from TCMM to construct the requisite knowledge graph for the model.

**Basic Seq2Seq**: The “Basic” model initializes embeddings arbitrarily without incorporating additional knowledge.

**CPMCP-based Seq2Seq**: The CPMCP database, which has the richest variety of relation types among TCM databases, is utilized for comparison. The ‘CPMCP-based’ model is pre-trained using relations from CPMCP.

**TCMM-based Seq2Seq**: The ‘TCMM-based’ model is pre-trained with a KG (supplementary figure S2) extracted from TCMM, which contains more diverse types of relations compared to CPMCP.

Regarding hyperparameters, the maximum epoch is set to 200 and an early stopping strategy is employed. The batch size is set to 128, the embedding dimension is 600, and the hidden dimension is set to 300. The Adam optimizer is chosen for optimization, with a learning rate of 0.001. Precision, Recall, and F1 score are utilized as evaluation metrics. TCMPR and KDHR are both multilabel classification models, within which the precision of top k recommended herbs has served as the evaluation metric in prior research. To standardize the evaluation metrics, appropriate probability thresholds are applied to limit the results of these models. Specifically, we identify the probability threshold that achieves the highest F1 score on the validation set, exploring within a range from 0.05 to 1.0 in increments of 0.01. Medicinal materials exceeding this threshold are regarded as possessing superior therapeutic effectiveness. Supplementary table S4 shows the results.

The experimental results show that the KDHR, TCMPR, and Basic Seq2Seq have similar performance in the F1 score. The introduction of additional TCM knowledge enables the model to achieve the best performance. Moreover, the model based on TCMM knowledge is better than the ‘CPMCP-based’ model in terms of Precision and F1 score for prescription generation, which means that a more comprehensive modernized TCM knowledge can help identify more symptomatic herbs, thus validating the potential of the TCMM database. The evaluation metrics are based on a statistical analysis of actual prescriptions extracted from the test dataset.

### 2.3 Multi-hop reasoning based TCM knowledge discovery

**Fig. 4:**
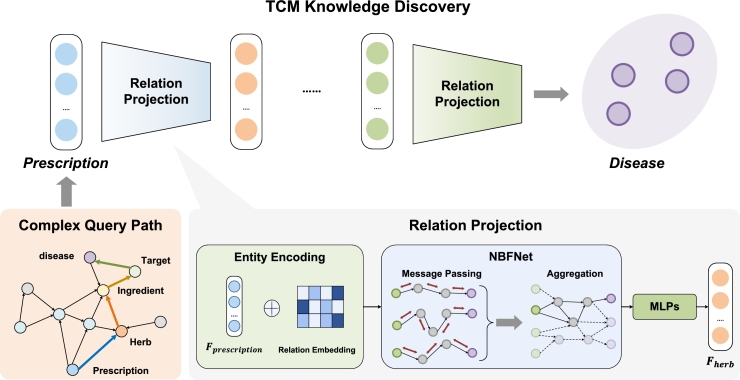
**TCM Knowledge Discovery Model** is employed to represent relations between entities within predefined complex query paths. Here, take prescription repositioning as an example, which utilizes the **complex query path**$prescription\overset{\;}{⟶}herb\overset{\;}{⟶}ingredient\overset{\;}{⟶}target\overset{\;}{⟶}disease$. **TCM Knowledge Discovery** takes the fuzzy set of the source entity *F*_*prescription*_ as input and iteratively performs relation projection operations to obtain the answer set *A*_*disease*_. **Relation Projection** initially merges the fuzzy set of the head entity in the corresponding triplet with the relation embedding, then utilizes the merged representation as input into the NBFNet for message passing and aggregation. Finally, the representation is mapped to the tail entity's fuzzy set through MLP layers and sigmoid.

Knowledge graph reasoning can infer new knowledge based on existing knowledge and has been widely applied in biomedical tasks such as drug-target interaction (DTI) and drug-drug interaction (DDI). However, the lack of direct relational data in the current TCM field makes it challenging for traditional knowledge graph reasoning methods to solve complex queries. Multihop reasoning, as a knowledge graph reasoning task, is primarily utilized to answer complex first-order logic (FOL) queries involving logical operations such as existential quantifier (∃), conjunction (∧), disjunction (∨), and negation (¬). Therefore, in this work, we introduce a multi-hop reasoning based pipeline in Fig. 4 to transform knowledge discovery into complex logical queries, discovering new knowledge through specific meta-paths.

#### 2.3.1 Data processing

To facilitate comparison with biological experiments, this work primarily concentrates on two tasks: Prescription-Disease and Symptom-Target. We extract relations from the database and construct a KG with task-relevant information specified based on expert knowledge. In contrast to the prescription generation task, which focuses on TCM-related attributes such as tropism and flavor, we retain the attribute entities of modern medicine, including biological process and pharmacological class, in order to investigate the correlation between TCM entities and modern medicine entities. Detail can be found in supplementary figure S3. We generate training data based on the method stated in [48], and introduce certain constraints, that filter out multiple self-related loops between entities and prohibit the appearance of bidirectional relations in the same path, to ensure the rationality of the path. In addition, logical operations, such as disjunction (∨), and negation (¬), are not considered based on the path features of downstream tasks. Furthermore, since the downstream tasks involve 4p and 5p queries, 4p and 5p data is utilized for training to enhance performance. To balance the performance of each query, the quantity of 4p and 5p is limited to one-tenth for other queries because the amount of answers for these tasks is significantly higher than others. The dataset is shown in supplementary table S5.

#### 2.3.2 Model description

GNN-QE [49], as the current state-of-the-art in the complex logical query task, is chosen to complete this task. This model predicts the fuzzy set of answer entities given the head entity and relation, where the elements in the set are all entities on the KG and are represented by a probability for the confidence of the tail entity.

GNN-QE initially transforms a FOL query into an expression of fundamental operations, enabling answer retrieval via expression execution. For example, FOL queries are decomposed into the expression (6).

(6) $$\text{FOL query:}\;q=c:\exists a,b:Consist\;of\;Herb(prescription,a)\;\;\wedge \mathrm{ConsistofIngredient}(a,b)\wedge \mathrm{AssociateTarget}(b,c)\text{Expression:}\;P_{\text{Associate}\;\;\text{Target}}(P_{\text{Consist}\;\;\text{of}\;\;\text{Ingredient}}\;\;\;\;\left(P_{\text{Consist}\;\;\text{of}\;\;\text{Herb}}(\{prescription\})\right))$$

$P_{r}(x)$ represents the tail entity fuzzy set measured with the relation r and the fuzzy set x of the head entity. {prescription} represents the entity sets related to the prescriptions.

A complex logical query on the KG attempts to predict the fuzzy set of the tail entity given the fuzzy set of the head entity x and the relation r. However, traditional KGC methods based on GNN concentrate on the relation projection between single entities, which is difficult to extend to large-scale fuzzy set prediction of the high time complexity. Therefore, NBFNet [50] is utilized to model relation projection in this study. Based on the generalized Bellman-Ford algorithm for single-source problems on graphs, NBFNet has been shown to perform well in the GNN-QE framework due to its lower complexity.

Following the NBFNet, we utilize the function to incorporate the probabilities in the head entity fuzzy set $h_{v}$ into the relation embedding, which together serves as the entity's initial representation. The entity representation is then input into a multi-layer GNN, integrating the structural information in the graph through message passing and message aggregation. The output of the final layer is then passed to the MLP layer, which predicts the fuzzy set of the tail entity using the sigmoid function. Based on the multi-hop relations in the path, multiple iterations are performed to ultimately predict the fuzzy set of answers. In equation (7), F represents the function that integrates the tail entity fuzzy set $P_{r}(x)$ of the previous hop and relation embedding. E(v) is the triplets of the training KG.

(7) $$h_{v}^{\left(t-1\right)}=F(P_{r}{\left(x\right)}^{\left(t-1\right)},r)h_{v}^{\left(t\right)}=\mathrm{Aggregate}\;\left(\mathrm{Message}\;\left(h_{v}^{\left(t-1\right)},E(v)\right)\right)P_{r}{\left(x\right)}^{\left(t\right)}=sigmoid\left(MLP\left({h_{v}}^{\left(t\right)}\right)\right)$$

According to [50], we choose binary cross entropy loss to train our model. Ans represents the answer set of the multi-hop query, V is the set of all entities and $y_{i}$ represents the probability of i in the final answer fuzzy set.

(8) $$\text{BCE Loss}=-\frac{1}{\left|\mathrm{Ans}\right|}\sum_{i\in \mathrm{Ans}}\log y_{i}-\frac{1}{\left|V-\mathrm{Ans}\right|}\sum_{i\in V-\mathrm{Ans}}\log\left(1-{\overset{ˆ}{y}}_{i}\right)$$

#### 2.3.3 Experimental study

Prior work [51], [52], [48], [49] of multi-hop reasoning mainly concentrated on evaluating the performance of 1p, 2p and 3p queries. However, this study focuses on long-path reasoning. To verify the impact of introducing 4p and 5p data to the model, we conduct an ablation study. $GNN-QE_{org}$ refers to the basic model, with the dataset presented in supplementary table S5 and $GNN-QE_{short}$ removes the 4p and 5p data from the trainset. To compare model performance, both models employ the same hyperparameters, which are shown in supplementary table S6.

The performance is measured by mean reciprocal rank (MRR). According to the results presented in supplementary table S7, the use of long path data demonstrates an enhancement in the performance for queries involving 3p, 4p, and 5p, while it will decrease the performance on short path queries. However, since TCM knowledge discovery requires the integration of intermediate information, it is necessary to have high performance in long-path reasoning.

## 3 Results

### 3.1 Modernized TCM research via web interface

**Fig. 5:**
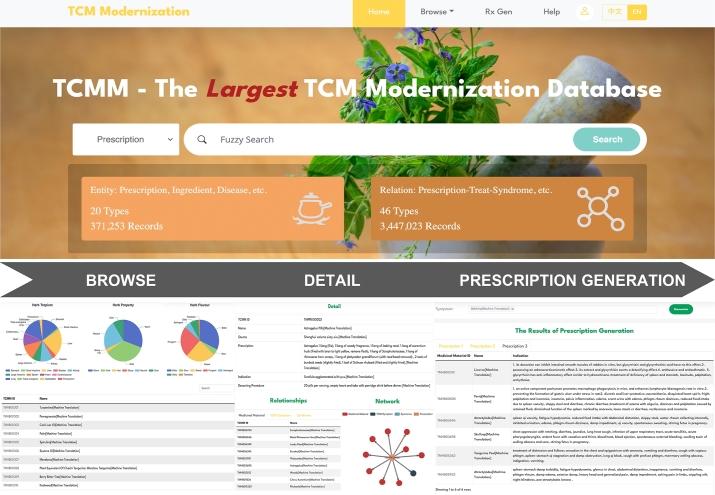
**Overview of Web Interface. Home Page** contains a navigation bar and a multi-entity search function. **Browse** shows the details and statistical results of each type of entity. **Detail** displays the entity's attributes and the associated relation types in the format of network and table. **Rx Gen** integrates the function of customized prescription generation. Users can get the top three prescriptions by entering the combination of the predefined 1402 symptoms.

TCMM presents a user-friendly website shown in Fig. 5, enabling users to effortlessly access comprehensive relations among various entities in both TCM and modern medicine. To enhance user experience, the website focuses on showcasing the nine most frequently used entities: prescription, medicinal material, ingredient, pathway, target, disease, TCM symptom, MM symptom, and syndrome.

The homepage of TCMM is equipped with a multi-entity search function, a concise description of the database, and a navigation bar filled with diverse functions. The search function is designed to support fuzzy search, allowing users to perform searches in Chinese, Pinyin, English, or using Alias names.

The Browse section is a collection of details and statistical results about the entities. Users can click on the ID within the details to view the relations of the entity. The Relation section is split into three parts: detail, network, and relations. The Detail primarily shows the entity's attributes, while the network and relation part displays the relation types associated with the entity in the form of a knowledge graph and a table, respectively.

A key function of TCMM is Rx Gen, which is used for online prescription generation. Users can freely combine any of the predefined 1402 symptoms to perform online reasoning. The website is programmed to automatically filter and display the top three prescriptions, each containing details about the medicinal material, thereby guiding the diagnosis and treatment in TCM.

### 3.2 Case studies

#### 3.2.1 TCM prescription generation

Beyond comparing model performance, it is necessary to validate the feasibility of generating prescriptions based on different types of modernized TCM knowledge. To validate the efficacy of the generated prescriptions, we randomly select various samples from the test set and compare the prediction results of the five models with ground truth. In the context of KDHR and TCMPR models, the predefined thresholds may result in all medicinal materials being filtered. To address this issue, we select the top 20 medicinal materials based on the probability of the result. In Table 2, herbs that appear in actual prescriptions are highlighted in green, herbs that do not appear in actual prescriptions but are symptomatic are highlighted in blue, and herbs not related to symptom are highlighted in red. Following the design of the model performance comparison experiment, ‘Basic’ represents a model without any additional knowledge incorporated. ‘CPMCP-based’ signifies a model that includes relations from the CPMCP database. The ‘TCMM-based’ model integrates relevant knowledge extracted from TCMM.

**Table 2:** **Case Study of Prescription Generation** is used to validate the efficacy of the generated prescriptions. Herbs used in actual prescriptions are marked in green, while herbs not in the prescriptions but symptomatically relevant are in blue, and those unrelated to symptoms are in red. ‘Basic Seq2Seq’ represents a model without any additional knowledge incorporated. ‘CPMCP-based Seq2Seq’ signifies a model that includes relations from the CPMCP database. The ‘TCMM-based Seq2Seq’ model integrates relevant knowledge extracted from TCMM. Besides, the results of TCMPR and KDHR are organized in descending order based on the probability values of the recommended medicinal materials, whereas the Seq2Seq model presents results in the form of inferred sequences.

Symptom	Ground Truth	TCMPR	KDHR	Basic Seq2Seq	CPMCP-based Seq2Seq	TCMM-based Seq2Seq
Inflammation	scutellaria baicalensis, rhubarb, phellodendron, scutellaria baicalensis, coptis chinensis, borneol, lycopi herba, folium hibisci mutabilis					
Bruising swelling	licorice, rehmannia glutinosa, clematidis armandii caulis, costus root, frankincense, red peony root, angelica sinensis, sappan wood, alisma orientalis, peach seed, chuanxiong, dipsaci radix, lycopi herba, cyathula root, cyperus rotundus, safflower, myrrh					
Epilepsy	licorice, spine date seed, rehmannia glutinosa, arisaema, atractylodes macrocephala, coptis chinensis, angelica sinensis, ginseng, figwort					
Headache	licorice, turmeric, white peony, angelicae pubescentis, cassia twig, parsnip					
Conjunctivitis	borax, bear bile					

Table 2 shows the comparison results between prescriptions generated by the models and ground truth. In comparison to KDHR and TCMPR, the Seq2Seq model demonstrates a significantly lower proportion of irrelevant medicinal materials in the results. This efficacy stems from the Seq2Seq model's capability to produce variable-length sequences, incorporating the sequence of herbs outputted at the previous step during the decoding process. Such a mechanism effectively reduces the likelihood of generating irrelevant medicinal ingredients. Conversely, multilabel classification models primarily focus on identifying all potentially relevant medicinal materials, to a certain extent overlooking the compatibility among herbs. Additionally, the pre-trained models show a greater tendency to predict symptomatic herbs than the model with random initialization, even if these herbs are absent from actual prescriptions. Meanwhile, the model trained with the TCMM KG is able to identify a greater amount of symptomatic herbs and fewer irrelevant herbs than the model trained with the CPMCP relations. This further validates the effectiveness of the abundant entity types and detailed relation types present in TCMM for the prescription generation task.

#### 3.2.2 TCM knowledge discovery

We evaluate the effectiveness of multi-hop reasoning in knowledge discovery for TCM modernization with a series of cases. To discover new insights, we only consider entities outside the training KG as the answer set. Meanwhile, for comparing the model outputs with actual experimental results, we choose prescription repositioning and symptom-related target prediction as downstream tasks, which have extensive experimental cases.

Table 3 shows the case study of prescription repositioning. To guarantee the validity of the path data, we choose $prescription\overset{\text{ consist of}}{⟶}herb\overset{\text{ consist of }}{⟶}ingredient\overset{\text{ associate }}{⟶}target\overset{\text{ associate }}{⟶}disease$ as the inference path based on the expert knowledge. We focus on five prescriptions with primarily concentrated components and select the top five answer entities based on their scores for analysis. The results demonstrate that the model's predictions of high-scoring outputs are highly consistent with the biological experimental results. Additionally, some modern medical insights distinct from TCM knowledge have been discovered. For instance, Salvia miltiorrhiza is primarily used in TCM to treat heart disease, but its active ingredient, Tanshinone IIA, has recently been shown to be effective in cancer treatment [53]. Another example is Panax notoginseng, whose main function is to promote blood circulation, and is frequently used to treat traumatic injuries. Its ingredient notoginsenoside R1 has been proven to be effective against pressure overload-induced cardiac hypertrophy, and the model associates it with aortic stenosis, the related symptom of pressure overload-induced cardiac hypertrophy. Therefore, TCMM can be utilized to predict the potential biological activities of natural products in various herbs, contributing to the development of natural medicinal chemistry and accelerating the discovery of investigational new drugs.

**Table 3:** **Case Study of Prescription Repositioning** shows the matching degree between the model's inference results and the experimental results of Prescription Repositioning task. If an item is marked in red, it indicates the consistency with the experimental results. ‘Publication’ shows the source of the experimental results.

Prescription	Disease	Publication
Galculus Bovis Capsules		[54]
	
	
	ecthyma 0.3	
	linitis plastica 0.3	

Danshen Granules		[53]
	
	
	neurotic depression 0.41	
	breast fibrocystic disease 0.4	

Patrinia Soup		[55]
	
	
	acanthoma 0.29	
	pleomorphic lipoma 0.29	

Panax Notoginseng Cream	diarrheal disease 0.35	[56]
	
	spindle cell neoplasm 0.32	
	sebaceous gland disease 0.31	
	dental abscess 0.31	
	dentin dysplasia type II 0.3	

Chuanbei Powder		[57]
	dentin dysplasia type II 0.3	
	fish eye disease 0.3	
	tarsal tunnel syndrome 0.29	
	deficiency anemia 0.28	

Inflammatory response as a common symptom has been extensively studied, and the understanding of its gene regulation mechanism significantly benefits modern medical diagnosis and treatment. Therefore, for the case study of symptom-related target prediction, we adopt $TCMsymptom\overset{\text{map}}{⟶}MMsymptom\overset{\text{ presented by }}{⟶}disease\overset{\text{ associate }}{⟶}target\overset{\text{ associate }}{⟶}target$ as the reasoning path and mainly analyze symptoms related to inflammation. The results in Table 4 show that the model can effectively predict symptom-related regulatory genes. For example, GABARAPL2, as a specific protein, is preferentially recognized by autoantibodies from early rheumatoid arthritis patients [58]. This kind of prediction may provide valuable knowledge for TCM researchers to identify the underlying molecular basis of patient symptoms.

**Table 4:** **Case Study of Symptom Related Target Prediction** shows the matching degree between the model's inference results and the experimental results of Symptom Related Target Prediction task. If an item is marked in red, it indicates the consistency with the experimental results. ‘Publication’ shows the source of the experimental results.

Symptom	Target	Publication
Rheumatoid Arthritis		[58]
	IQCJ-SCHIP1 0.34	
	
	SORT1 0.33	
	SH3GLB1 0.33	

Pneumonia		[59]
	STX12 0.29	
	NDUFA6 0.29	
	APEX2 0.28	
	VAMP4 0.28	

Tracheitis		[60]
	
	TFAP2C 0.4	
	FRMD1 0.39	
	SNTB2 0.39	

## 4 Discussion

Based on information regarding TCM prescription, symptom, target, and ingredient, numerous TCM databases have been developed using various data sources. Unfortunately, current efforts to clarify the pharmacological mechanisms of TCM are hindered by the absence of correspondence between TCM and Western medical knowledge. To address these issues, we conduct a study that integrates existing TCM and Western medical databases, refining the relations between the two fields through a standardized approach.

To enhance data quality, a rigorous screening and verification process is implemented for database entities. Specifically, we employ a data alignment process that filters entities based on specific rules, such as ID, name, description, and other relevant information. We also merge duplicate entities within the existing database to eliminate redundancy. For example, aliases are used to consolidate identical medicinal materials, and LLM is applied to merge semantically similar symptoms. These measures ensure that the database encompasses the majority of entities and relations in TCM and modern medicine, greatly enhancing its practicality in discovering modernized TCM knowledge. Moreover, the potential of the TCMM is validated from two perspectives. By integrating modernized TCM knowledge, the TCM prescription generation method is strengthened, resulting in prescriptions that are not only highly consistent with classical TCM prescriptions but also include new effective components to enhance the therapeutic effects of the original prescriptions. The field of TCM knowledge discovery is limited by the lack of relational data, leaving a gap in the use of computational methods to mine TCM knowledge. Existing methods typically rely on network pharmacology, with a singular and limited focus on specific prescriptions and diseases. The introduction of the TCMM database effectively bridges the gap between TCM and Western medical research, making AI-based, generalized TCM knowledge discovery possible. This study selects prescription repositioning and symptom-related target identification tasks with abundant cases for result validation. Experiments demonstrate that novel pathways consistent with the results of biological experiments can be discovered using TCMM knowledge, further proving the immense potential of TCMM in TCM knowledge discovery.

However, despite our experiments demonstrating the potential of TCMM knowledge, there are still some limitations. First, the database requires further improvements, particularly in relations of prescription. TCMM employs a hybrid approach that combines rules and LLM to extract relations among prescription, medicinal material, and symptom from text. Although the model achieves high accuracy in parsing prescriptions, extensive manual verification is still necessary to standardize the results, which will be addressed in future updates. Moreover, while TCMM data has bridged the knowledge between TCM and modern medicine, enabling the discovery of modernized TCM knowledge. However, long-path logical queries introduce a large number of random variables, inherently reducing the accuracy of the results. Additionally, the training data has not been completely validated by biological experiments, containing noise. Regarding interpretability, the validation of most high-probability inference paths is impeded by limitations in model performance and the absence of ground truth, rendering their accuracy unverifiable. Therefore, future research will continue to integrate the latest studies, improving the authenticity of the data, such as target and disease-related information. Additionally, graph data will be supplemented to shorten inference paths, thereby enhancing the confidence of the process for inferring new knowledge. Notably, the content of TCMM is for educational and scientific research purposes only and should not be utilized as a source of medical guidance or consultation. TCMM will be regularly updated to ensure its ongoing relevance and performance, accelerating the progress of modernized TCM research.

## Declaration of Competing Interest

The authors declare that they have no known competing financial interests or personal relationships that could have appeared to influence the work reported in this paper.

## Data Availability

Main data and related functions are publicly available through a web interface at https://www.tcmm.net.cn/. To obtain the complete data, please contact the corresponding author.

## Appendix A Supplementary material

MMC The supplementary materials integrate the tables and figures related to the method and result sections. These primarily include the construction details of the knowledge graph, as well as the data overview, hyperparameters, and comparison results of the deep learning models, which serve to support and validate the content of the article. MMC
