# Supplementary material for: TCMM: A unified database for traditional Chinese medicine modernization and therapeutic innovations
Source: Comput Struct Biotechnol J. 2024 Apr 15;23:1619–30. doi: 10.1016/j.csbj.2024.04.016 (PMC11047297; doi:10.1016/j.csbj.2024.04.016)
Supplement: MMC — The supplementary materials integrate the tables and figures related to the method and result sections. These primarily include the construction details of the knowledge graph, as well as the data overview, hyperparameters, and comparison results of the deep learning models, which serve to support and validate the content of the article. [file mmc1.docx]

**Supplementary Information**

**TCMM: A Unified Database for Traditional Chinese Medicine Modernization and Therapeutic Innovations**

*Zhixiang Ren^2^, Yiming Ren^2^, Zeting Li^2^, Huan Xu ^1,^**

*^1^ School of Public Health, Anhui University of Science and Technology, Hefei, Anhui Providence, 231131, China*

^2^ *Peng Cheng Laboratory, Shenzhen, Guangdong Province, China 518055*

*^*^ Co-corresponding author: Huan Xu, email:* [*2024078@aust.edu.cn*](2024078@aust.edu.cn)

Table of Contents

| **Table S1** | S2 |
| --- | --- |
| **Table S2** | S4 |
| **Table S3** | S8 |
| **Table S4** | S8 |
| **Table S5** | S8 |
| **Table S6** | S9 |
| **Table S7** | S9 |
| **Figure S1** | S10 |
| **Figure S2** | S11 |
| **Figure S3** | S12 |

**Table S1. Entity Information** encompasses the source, data volume, and detailed integration strategy for each type of entity in TCMM.

| **Entity Type** | **Count** | **Sources** | **Integration Strategy** |
| --- | --- | --- | --- |
| Tcm symptom | 1900 | CPMCP；SymMap | TCM symptoms come from CPMCP and SymMap. Due to some overlapping semantics in TCM Symptoms in the source databases, a large language model (LLM) is utilized to merge information from the source databases and combine it with manual verification to improve accuracy. Specifically, the semantic descriptions of TCM Symptoms are first generated using prompt engineering combined with GPT model and then convert the semantic descriptions into embedding representations using Sentence-BERT. To reduce computation, symptoms are grouped according to locus and property and only calculate similarity within groups. Through manual verification, 0.98 is selected as the similarity threshold, and symptoms with a similarity greater than the threshold are merged into one record. |
| Syndrome | 146 | SymMap | The data is extracted from SymMap and merge entries with the same Syndrome definition. |
| Mm symptom | 17079 | PharMeBINet;  CPMCP；  SymMap | The data is integrated from PharMeBINet, CPMCP and SymMap, and align with the MeSH ID. |
| Medicinal material | 8932 | CPMCP;  SymMap;  TCMBank | The records sharing the same HERB ID or SymMap ID are merged from three databases. If the merged records have different properties, combine the property descriptions and remove redundant information.  Furthermore, due to some herbs having multiple names, information on 4947 groups of herbs is extracted from <https://zhongyibaike.com> for cleaning alias data, thereby enhancing the recall rate in herb identification. |
| Prescription | 48043 | TCMID;  CPMCP | Prescriptions are integrated from CPMCP and TCMID, removing duplicate records. Moreover, the information on herbs from <https://zhongyibaike.com> is used to standardize the names of herbs in prescriptions. |
| Locus | 49 | CPMCP；  SymMap | Information about Symptom locus is extracted and aligned with Locus of the same meaning represented by a unified name, such as using "全身" to replace "通身" and "颈部" to replace "项". |
| Flavour | 13 | TCMBANK；CPMCP；  SymMap | The properties of herbs are divided into Tropism, Flavour, Property, and Toxicity. Properties with the same meaning are represented by a unified name through manual alignment. For Toxicity, there are some discrepancies among the three databases. To address this, a majority rule is adopted: if the majority is considered non-toxic, then it is classified as non-toxic, and further verification is conducted through manual screening. |
| Tropism | 13 | TCMBANK；CPMCP；  SymMap |  |
| Properties | 12 | TCMBANK；CPMCP；  SymMap |  |
| Toxicity | 4 | TCMBANK；CPMCP；  SymMap |  |
| Ingredient | 69816 | PharMeBINet;  CPMCP;  SymMap;  TCMBank | Since CAS ID, PubChem ID, and InChI Key cannot uniquely determine the molecule, the data is merged based on the same SymMap ID in TCMBank, CPMCP and SymMap but remove records in TCMBank pointing to multiple SymMap IDs. For example, TCMBANKIN057994 points to SMIT00245 and SMIT1456. For PharMeBINet, ingredient names are used to align entities. |
| Target | 76449 | PharMeBINet;  PrimeKG;  CPMCP;  SymMap;  TCMBank | NCBI ID and gene symbol are used to align data from SymMap, CPMCP, PrimeKG, and PharMeBINet, with gene symbol used to align TCMBank data. |
| Disease | 22365 | PharMeBINet;  PrimeKG;  CPMCP;  SymMap;  TCMBank | The data is integrated from the databases with keywords such as name, MONDO ID, OMIM ID, MeSH ID, Orphanet ID, and UMLS ID. Phenotypes, which are classified as Disease in TCMBANK, such as "height" and "cardiovascular issues" are filtered out. |
| Biological process | 28731 | PharMeBINet;  PrimeKG | To better understand the role of targets in disease processes, molecular function, biological process, and cellular component information are extracted from PrimeKG and PharMeBINet and aligned with GO ID. |
| Cellular component | 4191 | PharMeBINet;  PrimeKG |  |
| Molecular function | 11267 | PharMeBINet;  PrimeKG |  |
| Pathway | 3704 | PharMeBINet;  PrimeKG | The entities are integrated and aligned with specific IDs from the source databases. |
| Anatomy | 14033 | PharMeBINet;  PrimeKG |  |
| Side effect | 61729 | PharMeBINet;  PrimeKG |  |
| Pharmacologic class | 2777 | PharMeBINet |  |

**Table S2. Relation Information** encompasses the source, data volume, and detailed integration strategy for each type of relation in TCMM.

| **Relation Type** | **Count** | **Source** | **Integration Strategy** |
| --- | --- | --- | --- |
| Prescription-Contains-Medicinal Material | 320892 | CPMCP;  Manually Constructed | For prescription – medicinal material, to extract information about the dosage of medicinal materials from the unstructured descriptions in the database, ChatGLM is utilized to interpret the details of medicinal materials and their dosages within prescription descriptions. However, as the model's lack of training on similar data, the precision stands at only 57%. To enhance performance, we manually annotate a dataset for fine-tuning ChatGLM and randomly extract 200 prediction results for manual evaluation, achieving an accuracy of 96% and a recall rate of 76%. For incorrectly predicted prescriptions, further predictions are made using rule matching, achieving an accuracy rate of 98% under the same evaluation method. Ultimately, 320,892 prescription - medicinal material pairs are extracted, among which 264,728 relations included information on dosages. |
| Prescription-Treats-Symptom | 111155 | CPMCP;  Manually Constructed | For prescription – symptom/syndrome, the relation data partially originates from the CPMCP database, totaling 50,813 records, which only covers part of the prescriptions. To further supplement information for other prescriptions, TCMM extracts symptom and syndrome information from the indication and treatment attributes of prescriptions in the TCMID and CPMCP databases. Specifically, we use the symptom and syndrome entities obtained from CPMCP as keywords, match them with indication descriptions, and obtain an additional 60,342 prescription-symptom relations and 13,736 prescription-syndrome relations. |
| Prescription-Treats-Syndrome | 13736 | Manually Constructed |  |
| Ingredient-Associates-Target | 147810 | PharMeBINet; SymMap; CPMCP; TCMBank | For Ingredient - Target, Associate is considered as an ambiguous relationship and can be deemed redundant if a precise relationship exists. Therefore, Associate is mutually exclusive with the other three relationships. Since Downregulates and Upregulates have opposite meanings, if an entity pair exists with both relationships simultaneously, it is generalized as Associate. |
| Ingredient- Upregulates-Target | 46996 | PharMeBINet |  |
| Ingredient-Downregulates-Target | 46289 | PharMeBINet |  |
| Ingredient-Binds-Target | 1838 | PharMeBINet |  |
| Ingredient-Treats-Disease | 5489 | PharMeBINet | For Ingredient - Disease, semantically, Treats is mutually exclusive with the other two relationships. If it coexists with any one of relationships, it is considered as noise, and the entity pair will be deleted. |
| Ingredient-Induces-Disease | 7142 | PharMeBINet |  |
| Ingredient-Contraindicates-Disease | 418 | PharMeBINet |  |
| Target-Associates- Target | 374067 | PharMeBINet; PrimeKG | For Target - Target, Associate is mutually exclusive with the other two relationships. Furthermore, Associates and Covaries are considered as bidirectional relationships. Therefore, if both 'A - Covaries - B' and 'B - Covaries - A' coexist, they are deemed as redundant information, and only one of them is retained. |
| Target-Regulates-Target | 265667 | PharMeBINet |  |
| Target-Covaries-Target | 61605 | PharMeBINet |  |
| Disease-Associates- Target | 325428 | PrimeKG; SymMap; CPMCP; TCMBank | For Disease - Target, the relationships Associate, Downregulate, and Upregulate are mutually exclusive. For example, for the entity pair 'A - B', only one of these three relationships can exist. |
| Disease-Upregulates-Target | 7706 | PharMeBINet |  |
| Disease-Downregulates-Target | 7607 | PharMeBINet |  |
| Disease-Is a-Disease | 41950 | PharMeBINet | Because in PharMeBINet, "is a" represents an inclusion relationship, if both ‘A - is a – B’ and ‘B - is a – A’ exist simultaneously, these two triplets are deleted. For Resembles, while retaining only unidirectional relationships, triplets appearing in "Disease is a Disease" are removed. For example, if ‘A – Resembles – B’ and ‘ A - is a – B’ exist simultaneously, ‘ A - is a – B’ will be removed |
| Disease-Resembles-Disease | 537 | PharMeBINet |  |
| Ingredient-Resembles-Ingredient | 18407 | PharMeBINet | For Ingredient - Ingredient, since Resembles and Associates represent bidirectional information, if both ‘A – Resembles – B’ and ‘B - Resembles - A’ exist, only one is retained |
| Ingredient-Associates-Ingredient | 7498 | PharMeBINet |  |
| Medicinal Material-Tropism | 2174 | TCMBANK；CPMCP；  SymMap | In TCMBANK, CPMCP, and SymMap, the properties of herbs are divided into Tropism, Flavour, Property, and Toxicity. Properties with the same meaning are represented by a unified name through manual alignment. For Toxicity, there are some discrepancies among the three databases. To address this, a majority rule is adopted: if the majority is considered non-toxic, then it is classified as non-toxic, and further verification is conducted through manual screening. |
| Medicinal Material-Flavour | 1457 | TCMBANK；CPMCP；  SymMap |  |
| Medicinal Material-Property | 992 | TCMBANK；CPMCP；  SymMap |  |
| Medicinal Material-Toxicity | 142 | TCMBANK；CPMCP；  SymMap |  |
| TCM Symptom-Associates-Locus | 1488 | CPMCP;  SymMap | For TCM Symptom - Locus, information about Symptom locus is extracted and aligned, with Locus of the same meaning represented by a unified name, such as using "全身" to replace "通身" and "颈部" to replace "项" |
| Target-Associates-Biological Process | 161509 | PharMeBINet; PrimeKG | Integrate the data from 'INVOLVED_IN_GiiBP', 'ACTS_UPSTREAM_OF_OR_WITHIN_GauoowBP' in PharMeBINet and 'bioprocess_protein' in PrimeKG |
| Target-Associates-Cellular Component | 91617 | PharMeBINet | Integrate the relations 'LOCATED_IN_GliCC'，'IS_ACTIVE_IN_GiaiCC’, 'PART_OF_GpoCC','cellcomp_protein' from the database |
| Target-Associates-Molecular Function | 77526 | PharMeBINet; PrimeKG | Integrate the data from 'ENABLES_GeMF' in PharMeBINet and 'molfunc_protein' in PrimeKG |
| Ingredient-Associates-Biological class | 8977 | PharMeBINet | Integrate the relations 'ASSOCIATES_CHaBP', 'INCREASES_CHiBP', 'DECREASES_CHdBP' from the database |
| Ingredient-Associates-Molecular Function | 402 | PharMeBINet | Integrate the relations 'ASSOCIATES_CHaMF', 'DECREASES_CHdMF', ' INCREASES_CHiMF' from the database |
| Ingredient-Associates-Cellular Component | 38 | PharMeBINet | Integrate the relations 'ASSOCIATES_CHaCC', 'INCREASES_CHiCC', 'DECREASES_CHdCC' from the database |
| Ingredient-Belongs to-Pharmacologic Class | 1180 | PharMeBINet | The data of relations are extracted and merged from the source databases |
| Ingredient-Associates-Pathway | 604 | PharMeBINet |  |
| Ingredient-Causes-Side effect | 10931 | PharMeBINet |  |
| Ingredient-Might Causes-Side effect | 8748 | PharMeBINet |  |
| Disease-Presents-MM Symptom | 225690 | PharMeBINet; SymMap; CPMCP |  |
| Target-Associates-Pathway | 158225 | PharMeBINet; PrimeKG |  |
| Medicinal Material-Contains-Ingredient | 126857 | SymMap; CPMCP; TCMBank |  |
| Medicinal Material-Treats-TCM Symptom | 20371 | SymMap; CPMCP |  |
| Medicinal Material-Treats-Syndrome | 1863 | SymMap; CPMCP |  |
| Syndrome-Presents-TCM Symptom | 4919 | SymMap; CPMCP |  |
| TCM Symptom-Maps-MM Symptom | 2420 | SymMap; CPMCP |  |
| Anatomy-Expresses-Target | 526120 | PharMeBINet |  |
| Anatomy-Downregulates-Target | 102181 | PharMeBINet |  |
| Anatomy-Upregulates-Target | 97768 | PharMeBINet |  |
| Pathway-Leads to-Disease | 587 | PharMeBINet |  |

**Table S3. Medicinal Dosage Conversion** depicts the alignment method for dosage information in prescriptions. Measurement information within prescription descriptions has been standardized to grams (g) and milliliters (ml).

| 1 liang | 1 qian | 1 zhu | 1 fen | 1 li | 1 hao | 1 jin | 1 gongjin | 1 he | 1 dou |
| --- | --- | --- | --- | --- | --- | --- | --- | --- | --- |
| 31.25g | 3.125g | 1.3g | 0.3125g | 0.03125g | 0.003125g | 500g | 1000g | 20ml | 2000ml |

**Table S4.** **Prescription Generation Result.** Test results of the prescription generation model, which benefits from better database knowledge.

|  | **Precision** | **Recall** | **F1 score** |
| --- | --- | --- | --- |
| **TCMPR**  **KDHR**  **Basic seq2seq**  **CPMCP-based seq2seq**  **TCMM-based seq2seq** | 19.7  20.57  23.99  **25.27**  24.64 | 30.61  **30.98**  25.26  25.55  26.87 | 23.97  24.73  24.61  25.41  **25.71** |

**Table S5.** Statistics of different query types utilized in the multi-hop reasoning dataset

|  | **1p** | **2p** | **3p** | **4p** | **5p** |
| --- | --- | --- | --- | --- | --- |
| **Train**  **Test**  **Valid** | 249596  86900  86773 | 249596  7000  7000 | 249596  7000  7000 | 25000  7000  7000 | 25000  7000  7000 |

**Table S6. Hyperparameter of GNN-QE** are selected by the performance on the validation set.

| **Hyperparameter** | | **Values** |
| --- | --- | --- |
| **GNN** | Number of layers  Hidden dimensions | 4  32 |
| **MLP** | Number of layers  Hidden dimensions | 4  32 |
| **Traversal Dropout** | Probability | 0.25 |
| **Learning** | Batch size  Sample weight  Optimizer  Learning rate  Batch per epoch  Adv. temperature | 24  uniform across queries  Adam  5e – 3  300,000  0.2 |

**Table S7.** Test MRR result (%) of GNN-QE. ${GNN-QE}_{tcmm}$ is the model trained with 4p and 5p data, while ${GNN-QE}_{short}$ is not.

|  | **1p** | **2p** | **3p** | **4p** | **5p** |
| --- | --- | --- | --- | --- | --- |
| ${GNN-QE}_{tcmm}$  ${GNN-QE}_{short}$ | 13.9  **14.6** | 1.8  **1.9** | **1.8**  1.6 | **3.1**  2.9 | **3.9**  3.4 |



**Figure S1. LLM-based Prescription Knowledge Recognition** shows pipelines for processing two types of triplets: prescription-symptom and prescription-medicinal material. **(a) Indication Extraction from Prescriptions via Prompt Engineering Combined with LLM** employs Sentence BERT to measure the similarity between symptom descriptions generated by ChatGPT, facilitating the merger of synonymous symptoms. The prescription-symptom triplet is generated through keyword matching within prescription descriptions. **(b) Medicinal Material Dosage Extraction Based on LLM with Transfer Learning** involves fine-tuning ChatGLM with a manually curated dataset of prescription description-dosage pairs to enhance accuracy. The finetuned model is then utilized to transform prescriptions from TCMM into triplets that include dosage information.



**Figure S2. Knowledge Graph for Prescription Generation** displays the knowledge graph used by the prescription generation model for knowledge extraction. The attributes of medicinal material and prescription information are retained to characterize TCM compatibility principles. In addition, modern medical information such as target, disease, ingredient, and pathway are integrated to assist this task.



**Figure S3. Knowledge Graph for Knowledge Discovery** presents the knowledge graph utilized by the knowledge discovery model. To fully explore the correlation between TCM and Western medicine, only entities like anatomy and syndrome are removed, while all attribute information is preserved.
